# From Farm to Slaughter: Tracing Antimicrobial Resistance in a Poultry Short Food Chain

**DOI:** 10.3390/antibiotics14060604

**Published:** 2025-06-13

**Authors:** Andrea Laconi, Roberta Tolosi, Claudia Chirollo, Cristiana Penon, Giacomo Berto, Francesco Galuppo, Alessandra Piccirillo

**Affiliations:** 1 Department of Comparative Biomedicine and Food Science, University of Padua, Viale dell’Università 16, 35020 Legnaro, Italy; andrea.laconi@unipd.it (A.L.); roberta.tolosi@unipd.it (R.T.); 2 Department of Prevention, AULSS 8 Berica, 36100 Vicenza, Italy; claudia.chirollo@aulss8.veneto.it (C.C.); cristiana.penon@aulss8.veneto.it (C.P.); giacomo.berto@aulss8.veneto.it (G.B.); 3 Department of Prevention, AULSS 6 Euganea, 35131 Padua, Italy; francesco.galuppo@aulss6.veneto.it

**Keywords:** AMR, resistance genes, microbiota, poultry farm, short supply chain, poultry slaughterhouse

## Abstract

**Background**: Short food supply chains are commonly perceived as more sustainable and safer alternatives to conventional production systems, often linked to organic, free-range livestock practices. **Materials and methods**: This study investigates, for the first time, the distribution of antimicrobial resistance genes (ARGs) and characterizes the microbial communities’ composition, using 16S rRNA sequencing and real-time PCR, respectively. Eleven fecal, 76 slaughterhouse surface, 11 cecal, and 11 carcass samples, from 11 poultry farms belonging to the same short food chain, were analyzed in the study. **Results**: While cleaning and disinfection procedures appeared to reduce the bacterial load on slaughterhouse surfaces, diverse and potentially resistant bacteria, including genera such as *Staphylococcus* and *Streptococcus*, persisted both before and after slaughter. ARGs conferring resistance to high-priority critically important antimicrobials (HPCIAs), such as fluoroquinolones and third-generation cephalosporins, were frequently detected on carcasses, with *qnrS* (76.15%, 95%CI 68.02-84.28%) and *bla_CMY2_* (57.8%, 95%CI 48.38-67.22%) being the most prevalent. The slaughtering process emerged as a critical step for ARG dissemination via intestinal bacteria, such as genus *Lactobacillus*. Additionally, the detection of *mcr* genes and *bla_NDM_* on carcasses but not in the bird gut samples suggests possible anthropogenic contamination. **Discussion:** These findings highlight that the evisceration process, slaughterhouse environment, and personnel are all contributing factors in ARG spread and underscore the need for enhanced hygiene protocols and reduced gut ARG carriage in domestic birds to mitigate the risk for the consumer.

## 1. Introduction

The meat poultry industry represents one of the key and fastest-growing livestock sectors globally [1]. According to the Food and Agriculture Organization (FAO), the European Union (EU) is one of the main poultry meat producers worldwide, with an estimated output of 13 million tons per year [2]. Among European countries, Italy ranks as the fifth poultry meat producer, with approximately 1.33 million tons of meat produced in 2023 alone (https://www.unaitalia.com/, accessed on 1 April 2025). Indeed, poultry production represents the only self-sufficient Italian agricultural sector, producing 105.5% of the country’s need for poultry meat, and its total value has been estimated to be more than EUR 5.3 billion (https://www.unaitalia.com/, accessed on 1 April 2025).

The Italian poultry sector is mainly organized in vertically integrated companies, which control their entire production, from breeders and feed supply to the distribution of the final product. The integration, together with strict national legislations on biosecurity enforced by the official veterinary services spread all over the national territory, ensures the health and welfare of birds, as well as the safety of poultry meat [3]. Increased biosecurity and good farming practices have contributed to reducing the antimicrobial use (AMU) in conventional poultry farming [4]. AMU in livestock production has been linked to the emergence of resistant bacteria and determinants in the food production chain, posing a serious threat for human health, since they can be transferred to humans via dispersion into the environment (e.g., waterways, soil due to manure application) or by entering the food chain [5,6]. However, other factors (e.g., drinking water quality, biofilm, pests) beyond AMU can contribute to the spread of antimicrobial resistance (AMR) in the poultry production environment [7]. In this context, understanding the level of resistance towards highest priority critically important antimicrobials (HPCIAs) in the food chain should be prioritized, due to their significance in treating serious human infections and the high risk of resistance spread.

In recent years, the consumption of poultry meat produced outside of the conventional integrated companies has increased [8,9]. Consumers are increasingly attracted by agricultural practices that promote more sustainable products and alternatives to conventional farming, such as multifunctional farming, direct sales on the farm, and short food supply chains [10]. While these systems create a feeling of shared values between producers and consumers, their products are also often perceived as more ethical, safer, and superior in terms of organoleptic properties than those produced by the conventional production systems [11,12]. Short food chains represent a system that focuses on small-scale and local productions, minimizing the steps and the distance between producers and consumers while at the same time increasing the trustworthiness of the products and enabling small-scale entities to establish food supply chains beyond the great distribution. In this alternative system, small-scale farmers rely on local food chain infrastructures (e.g., slaughterhouse and markets), reducing competition among them, sharing resources and increasing negotiating power [13]. While this agricultural system may be beneficial for small-scale producers and represent a “greener” option to conventional farming, little is known about AMR, and the potential sources of contamination of resistant bacteria in domestic birds’ farms and slaughterhouses belonging to short food chains. Therefore, the aim of this research was to assess the prevalence of genes conferring resistance to HPCIAs, including carbapenems, third-generation cephalosporins, (fluoro)quinolones, and polymyxins, in poultry farms (i.e., chicken, guinea fowl, quail, duck, and pigeon farms), slaughterhouse surfaces, and carcasses within an Italian short food chain. Additionally, this study aimed to evaluate how these resistance genes relate to the microbiota of birds and their environment, in order to identify potential sources of antimicrobial resistance genes (ARGs).

## 2. Results

### 2.1. General Description of DNA Sequences

After the quality-filter step, removal of chimeric fragments and reads’ merging, a total of 2,514,642 reads were obtained, accounting for 9698 different features, with an average of 24,897 sequences per individual sample. Filtering by quality, all samples (n = 101) were used for the characterization of the microbial communities.

### 2.2. Microbial Load, Bacterial Communities’ Composition, and Diversity

The microbial load among sample types was measured as the mean log_10_ 16S rRNA copies/µL by real-time quantitative PCR (qPCR). The microbial load on pre-slaughter surfaces (surface_T1) was significantly lower compared to the other sample types, with the exception of carcasses (Figure S1). Notably, post-slaughter surfaces (surface_T2) showed a significantly higher microbial load than surface_T1 (*p* = 0.009, mean = 11.33 95% confidence interval (CI) 11.17–11.49 and mean = 10.58 95% CI 10.35–10.82, respectively).

**Figure 1:**
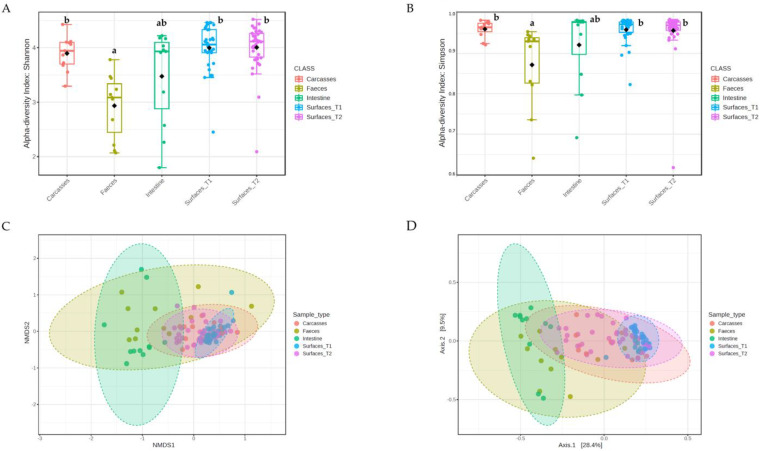
α-diversity within each sample using (**A**) Shannon’s and (**B**) Simpson’s indexes. Boxplots represent 25th to 75th percentiles; different letters indicate significant differences within the α-diversity indexes (*p* < 0.05). β-diversity between sample types according to Bray–Curtis distances using (**C**) Non-metric Multidimensional Scaling (NMDS) and (**D**) Principal Coordinate Analysis (PCoA).

16S rRNA sequencing was used to characterize the structure and composition of the microbial communities. The α-diversity, assessed at the Operational Taxonomic Unit (OTU) level using Shannon’s (Figure 1A) and Simpson’s (Figure 1B) indexes, was significantly lower in feces compared to carcasses (*p* = 0.0001), surface_T1 (*p* < 0.0001), and surface_T2 (*p* < 0.0001).

The analysis of β-diversity, explored using Permutational Multivariable Analysis of Variance (PERMANOVA) based on the Bray–Curtis dissimilarity measure, showed significant differences among all sample types, except between carcasses and surface_T2 (F = 0.75 and *p* = 0.639). However, fecal and cecal samples appeared more distant from other sample types (F = 3.68–26.35 and *p* = 0.001) than from one another (F = 2.48 and *p* = 0.016). Accordingly, Non-Metric Multidimensional Scaling (NDMS) and Principal Coordinate Analysis (PCoA) revealed a clear spatial separation between (i) fecal and cecal samples and (ii) carcasses and surfaces (Figure 1C, D). Furthermore, while some overlap can be observed between surface_T1, surface_T2, and carcasses, the two latter sample types showed a tendency to cluster closer to feces and ceca.

**Figure 2:**
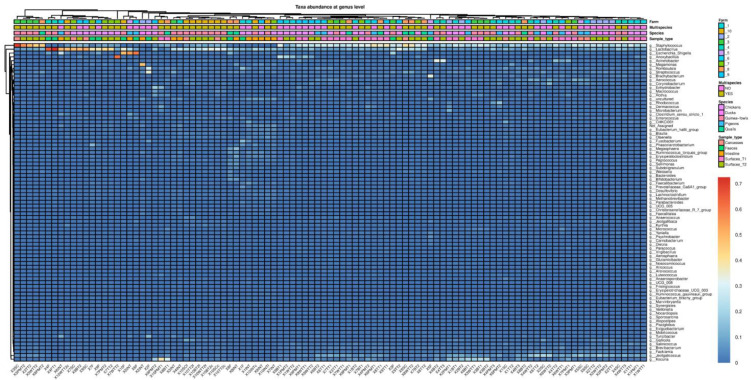
Heatmap representing the microbial community composition at the genus level of feces, surfaces, ceca and carcasses.

The phylum Firmicutes dominated the microbiota across all samples (Figure S2). However, bacteria belonging to the phyla Proteobacteria and Bacteroidota seemed more common in the microbiota of feces and ceca; meanwhile, Actinobacteriota were also abundant in samples collected from carcasses and slaughterhouse surfaces. The microbial community structure was further explored at the genus level. Core Microbiome Analysis identified 30 genera with prevalence and abundance higher than 20% and 0.01%, respectively, in all samples (Figure S3). Furthermore, Core Microbiome Analysis detected 15 genera shared among all sample types, such as *Clostridium*, *Enterococcus*, *Lactobacillus*, *Staphylococcus*, and *Streptococcus*. Although the genus *Lactobacillus* was a common feature among all sample types (Figure S3), the linear discriminant analysis (LDA) effect size method (LEfSe) showed that bacteria belonging to this genus were more abundant in feces, ceca, and carcasses (LDA score = 4.99) compared to surfaces (Figure S4). Similarly, the genus *Escherichia/Shigella* showed higher abundance in fecal and cecal samples (LDA = 4.88) than in the other sample types. In contrast, *Staphylococcus, Aerococcus*, and *Jeotgalcoccus* seemed to characterize the microbiota found on carcasses and surfaces (LDA = 5.08, LDA = 4.62, and LDA = 4.15, respectively). Accordingly, in the heatmaps at the genus level, samples obtained from the slaughterhouse surfaces and carcasses tended to cluster together (Figure 2).

### 2.3. ARGs Prevalence and Relative Abundance

**Figure 3:**
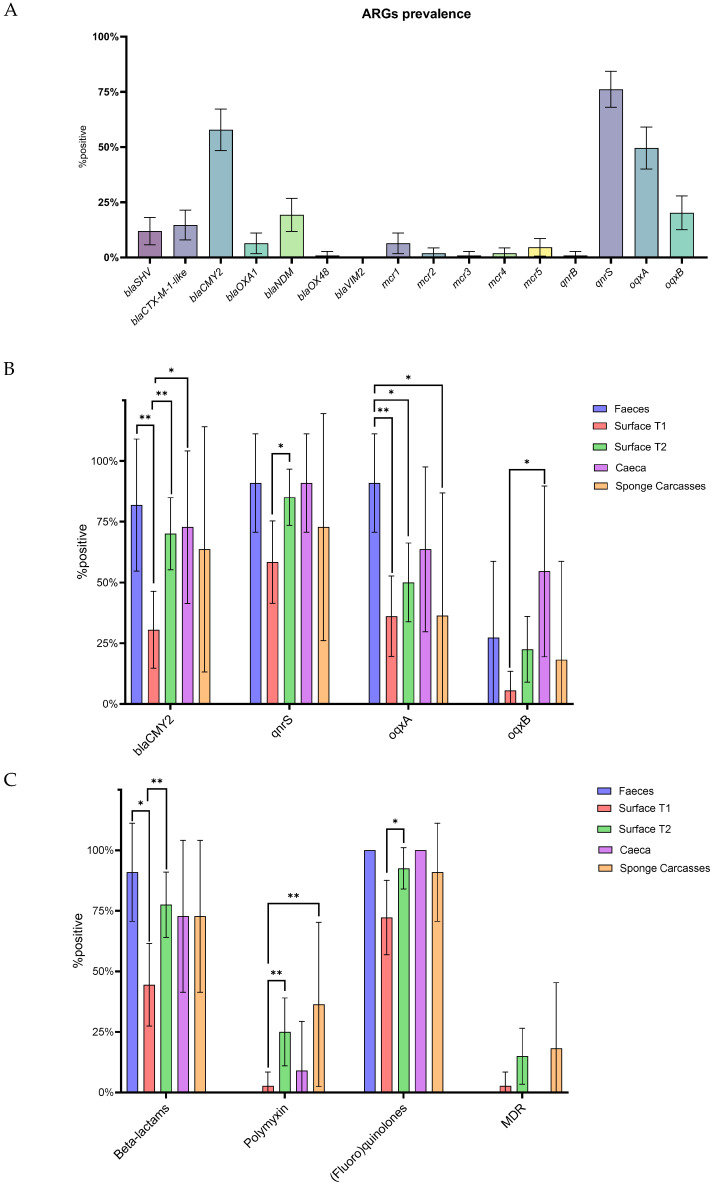
Prevalence of target ARGs in feces, ceca, carcasses, and surfaces. (**A**) Overall prevalence of antimicrobial resistance genes (ARGs). (**B**) Prevalence of ARGs according to sample type. (**C**) Prevalence of class-resistance and MDR according to sample type. *p* < 0.05 shown as * and *p* < 0.01 as **. Whiskers represent 95% CI.

The prevalence of ARGs in feces, ceca, carcasses, and surfaces was assessed by real-time PCR. Out of the sixteen investigated genes, only *bla_VIM2_* went undetected, while the remaining were identified in at least one sample. The *qnrS* gene was the most prevalent gene (76.15%, 95% CI 68.02–84.28%), followed by *bla_CMY2_* (57.8%, 95% CI 48.38–67.22%) and *oqxA* (49.54% 95% CI 40.0–59.08%). The prevalence of the other genes ranged from 20.18% (95% CI 12.53–27.84%) for *oqxB* to 0.92% (95% CI 0.0–2.74%) for *bla_OXA48_*, *mcr-3*, and *qnrB* (Figure 3A).

When considering each individual antimicrobial class, 87.16% (95% CI 80.77–93.54%) of the samples tested positive for at least one gene conferring resistance to (fluoro)quinolones, 66.97% (95% CI 58.0–75.94%) to β-lactams, and 14.68% (95% CI 7.93–21.43%) to polymyxins. Only 8.26% (95% CI 3.01–13.51%) of the samples showed a multi-drug resistance profile (i.e., resistance to three antimicrobial classes) (Figure S5). Differences in the distribution of ARGs among sample types were observed (Figure 3B). For instance, the *bla_CMY2_* gene was more prevalent in feces (*p* = 0.0044, odd ratio = 10.23, 95% CI 1.871–50.48), ceca (*p* = 0.0176, odd ratio = 6.06, 95% CI 1.29–23.29) and surface_T2 (*p* = 0.0011, odd ratio = 5.3, 95% CI 2.0–13.61) compared to surface_T1. Binomial analysis also revealed that *qnrS* was more prevalent in surface_T2 compared to surface_T1 (*p* = 0.0014, odd ratio = 4.05, 95% CI 1.32–12.51). Meanwhile, the *oqxA* gene was detected with higher frequencies in fecal samples than in carcasses (*p* = 0.0237, odd ratio = 17.5, 95% CI 1.48–212.0), surface_T1 (*p* = 0.0018, odd ratio = 17.69, 95% CI 2.19–199.0) and surface_T2 (*p* = 0.0173, odd ratio = 10.0, 95% CI 1.31–113.4). The *oqxB* gene was more prevalent in the ceca of slaughtered birds compared to surface_T1 (*p* = 0.0014, odd ratio = 18.6, 95% CI 2.86–99.22). Interestingly, none of the fecal samples were positive for any *mcr* genes (*mcr-1* to *mcr-5*). Surface_T1 samples were found to be less likely to harbor genes conferring resistance to β-lactams compared to feces (*p* = 0.0129, odd ratio = 12.5, 95% CI 1.58–141.6) and surface_T2 (*p* = 0.0044, odd ratio = 4.31, 95% CI 1.65–11.54), as well as genes conferring resistance to polymyxins compared to carcasses (*p* = 0.008, odd ratio = 20, 95% CI 2.37–248.9) and surface_T2 (*p* = 0.0076, odd ratio = 11.67, 95% CI 1.71–130.0), and genes conferring resistance to (fluoro)quinilones compared to surface_T2 (*p* = 0.0308, odd ratio = 4.74, 95% CI 1.13–16.97) (Figure 3C). No differences in MDR profile were observed among the sample types.

### 2.4. Association Between Microbial Communities and ARGs

The association between the relative abundance of genera forming the core microbiome (≥20% prevalence and ≥0.01% abundance) and ARGs (≥10% prevalence) was assessed using multiple logistic regression. Four genera were found to be positively associated with the occurrence of at least one ARGs, with *Aerococcus* genus being associated with more than one gene. In detail, *Aerococcus* and *Escherichia/Shigella* genera were associated with *bla_CTX-M-1like_* (*p* = 0.0153, odd ratio (O.R.) = 6.25, 95% CI O.R. = 3.99–41.26 and *p* = 0.0281, O.R. = 3.68, 95% CI O.R. = 1.35–14.71, respectively), while *Lactobacillus* was strongly associated with *bla_CMY2_* (*p* = 0.0047, O.R. = 36.05, 95% CI O.R. = 3.56–548.4). Furthermore, the prevalence of *qnrS* gene was associated with the abundance of *Aerococcus* (*p* = 0.025, O.R. = 16.66, 95% CI O.R. = 1.72–259.9) and Anoxybacillus (*p* = 0.0031, O.R. = 6.02, 95% CI O.R. = 2.06–23.54).

## 3. Discussion

Short food supply chains are commonly regarded as more sustainable alternatives to conventional farming systems and are often associated with free-range organic livestock practice by consumers [10]. In the present study, culture-independent approaches, such as 16SrRNA sequencing and real-time PCR, were used to investigate, for the first time, the potential sources of antimicrobial resistance determinants and bacteria in a poultry short production chain, from the farms to the slaughterhouse.

The microbial community compositions of fecal and cecal samples resembles those previously identified in free-range organic poultry farms, with *Escherichia/Shigella* and *Lactobacillus* genera being the most abundant [7]. While cleaning and disinfection procedures seem to reduce the total microbial load, pre-slaughter surfaces still harbored a rich and diverse bacterial community. Among the genera identified, several (e.g., *Staphylococcus* and *Streptococcus*) are known to produce biofilm on abiotic surfaces, carry disinfectant resistance genes, and be able to activate SOS response genes, which may explain their persistence despite routine disinfection procedures [14,15]. A notable shift in microbial composition was observed on post-slaughter surfaces, likely due to contamination with intestinal bacteria (e.g., *Lactobacillus*) during the evisceration process [15,16,17]. Interestingly, the microbiota of carcasses and post-slaughter surfaces tended to overlap (β-diversity), suggesting that slaughterhouse surfaces may serve as a bridge for the transfer of intestinal microbes to carcasses [17]. In contrast to other studies conducted in poultry slaughterhouses, pathogenic bacteria, such as *Pseudomonas*, *Salmonella* spp., and *Campylobacter* spp., were not among the most abundant genera identified on carcasses and slaughterhouse surfaces [18]. However, the high abundance of bacteria belonging to the genus *Staphylococcus* in pre- and post-slaughter surfaces, as well as carcasses, represents a critical point and a potential concern for the health of both consumers and workers, as further discussed later [19].

Although antimicrobial use is widely recognized as a key driver of AMR emergence in livestock, several studies have reported the presence of ARGs in rearing systems where antimicrobials are rarely, if ever, used. This is because factors such as drinking water quality, biofilms, and pests can contribute to the spread and maintenance of AMR in poultry farms. [7,20,21,22]. Consistent with these findings, this study detected genes conferring resistance to HPCIAs (i.e., carbapenems, third-generation cephalosporins, (fluoro)quinolones, and polymyxins) in the feces and ceca of untreated birds, as well as on their carcasses and slaughterhouse surfaces. Notably, all samples collected at the farm, all intestinal contents collected at the slaughterhouse, and nearly all carcasses (90.91%, 95% CI: 70.65–100%) were found to be positive for at least one gene associated with (fluoro)quinolone resistance (i.e., *oqxA*, *oqxB*, *qnrS*, and *qnrB*). Similarly, high prevalence of β-lactams resistance genes (>70%) was found on carcasses and post-slaughter surfaces. These findings partially agree with previous observations reporting a high prevalence of genes conferring resistance to β-lactams in carcasses from free-range poultry farming [23]. In contrast to the present study, Ferri et al. (2023) [23] did not detect any genes conferring resistance to (fluoro)quinolones and carbapenems in antibiotic-free and conventional broiler farms. While these contrasting results may be due to differences in the species investigated, and therefore to different agricultural practices, they should be interpreted with caution. Notably, Ferri et al. (2023) [23] investigated only one (fluoro)quinolone resistance gene (i.e., *parC*). Moreover, they employed an end-point PCR approach, which is a less sensitive method compared to the one used in the present study. However, the detection of HPCIAs resistance genes on poultry carcasses and within the slaughterhouse environment raises important public health concerns, as these may act as reservoirs and transmission routes for AMR from animals to humans through the food chain [24,25].

Notably, the prevalence of ARGs, along with the total bacterial load, was significantly lower (*p* < 0.05) on pre-slaughter surfaces compared to post-slaughter surfaces across all the examined antimicrobial classes. Although cleaning and disinfection procedures appeared to reduce the overall load of ARGs and bacteria on the slaughterhouse surfaces, this effect was not consistent across all investigated ARGs. For instance, the persistence of *qnrS* in the slaughterhouse environment represents one of the main issues identified in the present study. While its prevalence significantly increased on surfaces following evisceration, it was also found in almost 60% (58.33, 95% CI 41.42–75.25%) of pre-slaughter surfaces. Given its presence in over 90% (90.91%, 95% CI 70.65–100%) of feces and ceca, the high occurrence of *qnrS* on carcasses likely results from multiple contamination events, both from gut bacteria released during slaughtering and from bacteria persisting on processing surfaces [16,17,26]. The ability of *qnrS*-carrying bacteria to survive on slaughterhouse surfaces may be linked to the localization of this gene on mobile genetic elements (MGEs) also carrying disinfectant resistance genes [27]. Another concerning finding of this study was the high prevalence (63.64 95% CI 29.74–97.53%) of *bla_CMY2_*, a gene conferring resistance to third-generation cephalosporin, on carcasses. However, while the presence of *qnrS* seems to rely on multiple contamination events, the high prevalence of the *bla_CMY2_* gene on carcasses seems to be primarily due to the dissemination of resistant bacteria originating in the birds’ microbiota, while the contribution of bacteria persisting on the slaughterhouse surfaces seems to be secondary [16]. In fact, the positive association between *bla_CMY2_* and the *Lactobacillus* genus, which dominated the microbiota of ceca, post-slaughter surfaces, and carcasses, seems to suggest that the contaminations likely occurred during the evisceration process. Although *Lactobacillus* are commonly considered harmless bacteria, they may act as reservoir and spreaders of β-lactams resistance determinants across multiple bacterial species, including pathogens [28,29].

These findings support the hypothesis that viscera removal during slaughter is a critical factor contributing to the dissemination of ARGs from intestinal bacteria to carcasses [16,17]. However, intestinal contents and persistent bacteria in the slaughterhouse environment may not be the sole contamination sources. Personnel may also represent a potential source of ARG transmission to carcasses [17]. For instance, the prevalence of *mcr* genes, which confer resistance to polymyxins, significantly increased in carcasses and post-slaughter surfaces compared to pre-slaughter surfaces. However, no *mcr* genes were found in feces and only one cecal sample tested positive for *mcr-1*. This distribution pattern suggests a possible anthropogenic origin of these resistance determinants. Indeed, future research is needed to confirm this hypothesis, as it was beyond the scope of the present study. Similarly, carbapenems resistance gene *bla_NDM_* was absent in fecal samples but was consistently detected in the slaughterhouse environment, peaking in prevalence on carcasses (36.36%, 95% CI 2.47–70.26%). Considering that both carbapenems and polymyxins are last-resort drugs for treating multi-resistant bacterial infections [30], enhanced efforts (e.g., improved sanitation protocols and stricter hygiene practices among workers) are warranted to reduce the prevalence of these ARGs on carcasses. Although no correlation was found between the resistance determinants investigated and the *Staphylococcus* genus (*p* > 0.05), the higher abundance of these bacteria on carcasses and surfaces, compared to feces and ceca, further supports the role of the slaughterhouse environment and personnel as potential sources of contamination [19].

Conversely, some resistance genes appear to be primarily restricted to the birds and their farm environment. For instance, *oqxA*, which confers resistance to (fluoro)quinolones, was detected in nearly all farm samples (90.91%, 95% CI 70.65–100%); however, it was present in about a third (36.36%, 95% CI 2.47–70.26%) of carcasses. Previous research has shown that *oqxA* can persist in litter, manure, and soil, yet appears less stable on abiotic surfaces subjected to periodic disinfection procedures [7,31]. This behavior may account for the limited transfer of *oqxA* from the birds’ gut to the carcasses via the slaughterhouse surfaces. A similar pattern was also observed for three genes conferring resistance to β-lactams, i.e., *bla_SHV_*, *bla_CTX-M-1-like_*, and *bla_OXA_*. Notably, logistic regression analysis revealed a positive association between *bla_CTX-M-1-like_* and *Escherichia/Shigella* genus, which was more abundant in fecal and ceca samples compared to the other sample types. The lower abundance of these bacteria on the slaughterhouse surfaces may have limited the spread of *bla_CTX-M-1-like_* from the birds’ gut to the carcasses. This finding, combined with the high prevalence of *bla_CMY2_* in association with a high abundance of *Lactobacillus*, suggests that the presence of resistance genes on carcasses may be primarily attributed to contamination through inadvertent leakage of gut contents directly on the carcasses, or indirectly on the slaughterhouse surfaces, rather than to horizontal gene transfer (HGT), even for genes associated with MGEs [16].

Overall, the distribution of ARGs varied across sample types, reflecting differences in their persistence within the slaughterhouse environment, dissemination dynamics during processing, and ultimately multiple contamination patterns on carcasses. Notably, different distribution patterns and dynamics were observed among genes conferring resistance to the same antimicrobial class. Although routine cleaning and disinfection procedures appear to reduce both microbial load and ARG prevalence on surfaces, resistant determinants, such as *qnrS*, proved capable of persisting in the slaughterhouse environment. Moreover, the evisceration process itself seems to be a key contributor to carcass contamination, highlighting the need for optimized processing protocols and reducing the intestinal load of resistant bacteria and resistance genes in rearing birds. Indeed, reducing the prevalence of antimicrobial resistant bacteria and determinants in the birds’ gut microflora may pose a lower risk for consumers [32]. Although this study was limited to only one poultry short food supply chain, it contributes to improving the knowledge on the structure of the microbiota and the distribution of ARGs on carcasses, and on how they are affected by the entire slaughtering process. Future studies should focus on two key areas: (1) investigating differences in AMR load among various poultry species, and (2) elucidating the role played by different slaughterhouse surfaces in carcass contamination. This includes identifying which surfaces are more likely to contribute to carcass contamination with resistant determinants and bacteria. These findings may help identify critical points during the slaughtering process and improve slaughtering protocols, as well as cleaning and disinfection procedures.

## 4. Materials and Methods

### 4.1. Sampling Procedure

Eleven small-scale poultry farms, belonging to the same short food chain and processing their birds at the same slaughterhouse, were included in this study. The farms included in this study were located in Northeastern Italy and comprised mono-species (n = 5) and multi-species farms (n = 6). During the farm visit for samples collection, an ad hoc questionnaire (File S1) was compiled. The characteristics and demographics of the farms are reported in Table 1.

Samples were collected from March to July 2024. The farms were visited once, at the end of the rearing cycle (T0), i.e., from one to three days before slaughtering. In each farm, at least 25 g of fecal droppings were collected from ten randomly selected locations of a barn using a sterile spatula and then placed into a sterile tube. At the slaughterhouse, work surfaces (i.e., conveyor belt, plucking machine, water tank, and trolley) and carcasses (n = 10) were sampled using sterile sponges soaked in buffered peptone water (BPW). Meanwhile, the cecum (the distal part of the ileum in pigeons) was aseptically removed from the same ten previously sampled carcasses. The slaughterhouse was visited ten times (because birds from two farms were slaughtered on the same day), specifically whenever a batch of birds coming from the selected farms was processed. During each visit, the surfaces were sampled twice: prior to the first slaughter of the day (surface_T1) and after processing the birds from the sampled farms (surface_T2). All samples were immediately stored at −80 °C until DNA extraction. In total, 11 fecal, 76 slaughterhouse surface, 11 cecal, and 11 carcass samples, for a total of 109 samples, were collected during this study.

### 4.2. DNA Extraction

Fecal samples were pre-treated as previously described [7]. Briefly, 10 g of feces were mixed by vortexing (1 min) with 10 mL of Phosphate-Buffered Saline (PBS) and then centrifuged at 4000 rpm for 10 min at 4 °C. DNA was extracted from 250 mg of the resulting pellet using the DNeasy PowerLyzer PowerSoil^®^ kit (Qiagen, Hilden, Germany) following the manufacturer’s instructions. Surface and carcass sponges underwent the same pre-treatment. In detail, each sponge was placed in a sterile filter-bag (280 µm pore size), to which 50 mL of PBS was added and mixed for 7 min using a Stomacher^®^ (VWR International, Allentown, PA, USA). The filtered liquid was centrifuged at 4000 rpm for 10 min at 4 °C. Cecal content was collected from each cecum (ten per farm) using cotton sterile swabs (one per cecum). Each swab was eluted in 1 mL of PBS and mixed for 90 s at 25 hertz using TissueLyser^®^ (Qiagen) and the supernatant was collected. Supernatants obtained from samples belonging to the same sampling unit (i.e., birds from the same farm) were pooled together and centrifuged at 4000 rpm for 10 min at 4 °C. DNA was extracted from the pellet obtained from sponges and cecal swabs using the DNeasy PowerSoil Pro^®^ kit (Qiagen). DNA quantity was assessed using an Agilent 2100 Bioanalyzer (Agilent Technologies, Palo Alto, CA, USA).

### 4.3. Microbial DNA Load, 16S rRNA Gene Amplification, Sequencing, and Data Analysis

The 16S rRNA gene copy number in samples was quantified by qPCR paired with melting curve analysis, as previously described [33]. Briefly, each sample was tested in triplicate using PowerUp™ SYBR^®^ Green Master Mix (Thermo Fisher Scientific, Waltham, MA, USA) in a LightCycler^®^480 Roche (Roche, Basel, Switzerland) real-time platform. NGS-based sequencing of the V3–V4 regions of the 16S rRNA gene was employed to explore the microbiome of each sample. Libraries were prepared as previously described [34] and sequenced using the Illumina MiSeq sequencing platform (San Diego, CA, USA) with a 2 × 300 bp paired-end approach. Prior to sequencing, libraries were quantified using the Qubit 2.0 Fluorometer (Invitrogen, Life Technologies, Monza, Italy) and DNA integrity was assessed using an Agilent 2100 Bioanalyzer (Agilent Technologies, Palo Alto, CA, USA). Due to poor quantity/integrity, eight libraries were excluded from this study. Data analysis and taxonomic assignment of 16S rRNA sequences were carried out using DADA2 package (v1.26) within the Quantitative Insights into Microbial Ecology 2 (QIIME2 v2023.9) software [35,36] and the SILVA-Naive Bayes sklearn trained database [37], respectively. The structure of the microbial communities was explored using a heatmap (pheatmap v1.0.12 package within Rstudio v2024.12.1). The microbial diversity within each sample type (α-diversity) was assessed using Shannon’s and Simpsons’ indexes, while differences among sample types (β-diversity) were assessed using PERMANOVA based on the Bray-Curtis dissimilar measure. β-diversity was then visualized with PCoA and NDMS. Core Microbiome Analysis was used to identify taxa shared among sample types. Taxa likely to explain differences between sample types were identified using LEfSe. Data analyses and visualization were carried out using the online software MicrobiomeAnalyst (https://www.microbiomeanalyst.ca, accessed on 20 May 2025). The raw sequence reads were deposited in the NCBI Short Read Archive under accession number PRJNA1260470.

### 4.4. Real-Time PCR Analysis of Antimicrobial Resistance Genes (ARGs)

Gene-specific real-time polymerase chain reaction (real-time PCR) assays, paired with melting curve analysis, were used for detecting sixteen ARGs conferring resistance to three antimicrobial classes, namely, β-lactams (i.e., *bla_SHV_*, *bla_CTX-M-1like_*, *bla_CMY-2_*, *bla_OXA-1_*, *bla_OXA-48_*, *bla_VIM-2_*, and *bla_NDM_*), polymyxins (i.e., *mcr-1*, *mcr-2*, *mcr-3*, *mcr-4*, and *mcr-5*), and (fluoro)quinolones (i.e., *oqxA*, *oqxB*, *qnrS*, and *qnrB*), as previously described [38]. The samples were tested in triplicate for each gene using a PowerUp™ SYBR^®^ Green Master Mix (Thermo Fisher Scientific) in a LightCycler^®^480 Roche (Roche) real-time platform. Primers’ sequences, optimal concentration, annealing and melting temperatures, and positive controls used in this study are reported in Table S1.

### 4.5. Statistical Analysis

The non-parametric Kruskal–Wallis test with Dunn’s test for multiple comparisons were used to assess differences in microbial DNA load (adjusted mean log_10_ 16S rRNA copies/µL) among sample types. Differences in the occurrence of each ARG (binary outcome variable), class-level resistance (i.e., at least one ARG per antimicrobial class) and multi-drug resistance (i.e., resistance to at least three antimicrobial classes) across sample types were assessed using Fisher’s exact test. To investigate the potential associations between the relative abundance of microbial taxa at the genus level (minimum prevalence level of 20% and minimum abundance of 0.01% in all samples) and ARGs (minimum prevalence level of 10% in all samples), the relative abundances of taxa (dependent variables) were jointly regressed on the presence/absence of ARGs (independent variables) using multiple logistic regression within GraphPad Prism (v. 10.4.1).

## Figures and Tables

**Table 1:** Characteristics of poultry farms included in this study.

Variable	Number (n)	Percentage
**Number of barns**		
≤2	7	63.64%
3–4	1	9.09%
≥5	3	27.27%
**Surface (m^2^)**		
<100	2	18.18%
100–1000	4	36.36%
>1000	1	9.09%
Unknown	4	36.36%
**Multispecies**		
Yes	7	63.64%
No	4	36.36%
**Species**		
Chickens	4	36.36%
Quails	1	9.09%
Ducks	1	9.09%
Guinea fowls	3	27.27%
Pigeons	2	18.18%
**Number of birds**		
<300	4	36.36%
300–1000	3	27.27%
>1000	3	27.27%
Unknown	1	9.09%
**Number of birds slaughtered**		
<50	4	36.36%
50–100	3	27.27%
>100	4	36.36%
**Water source**		
Water main	9	81.82%
Water well	2	18.18%
**Water distribution system**		
Manual	4	36.36%
Automatic	6	54.55%
Unknown	1	9.09%
**Feed source**		
Purchased	6	54.55%
Home made	1	9.09%
Both	4	36.36%
**Feed distribution system**		
Manual	9	81.82%
Automatic	2	18.18%
**Ventilation system**		
Manual	10	90.91%
Automatic	1	9.09%
**Antimicrobial treatments during cycle**		
Yes	1	9.09%
If yes, specify;	Trimethoprim/sulfamethoxazole	
No	10	90.91%

## Data Availability

Sequencing data were deposited on NCBI and are publicly available (PRJNA1260470).

## Supplementary Materials

The following supporting information can be downloaded at: https://www.mdpi.com/article/10.3390/antibiotics14060604/s1, Figure S1: Violin plots reporting the microbial load measured as adjusted mean log10 16S rRNA copies/µL for each sample type; Figure S2: Relative abundance of phyla in all samples; Figure S3: Core Microbiome Analysis; Figure S4: Linear discriminant analysis (LDA) effect size method (LEfSe); Figure S5: Overall prevalence of class-level and multi-drug resistance (MDR) in all samples. File S1: Data Collection and Sample Accompaniment Form; Table S1: list of primers used in qPCR assays for the detection of the selected ARGs.
