# Supplementary material for: From Farm to Slaughter: Tracing Antimicrobial Resistance in a Poultry Short Food Chain
Source: Antibiotics (Basel). 2025 Jun 13;14(6):604. doi: 10.3390/antibiotics14060604 (PMC12190163; doi:10.3390/antibiotics14060604)
Supplement: Supplementary file 1 [file antibiotics-14-00604-s001.zip › Figure S1.pptx]

## Slide 1
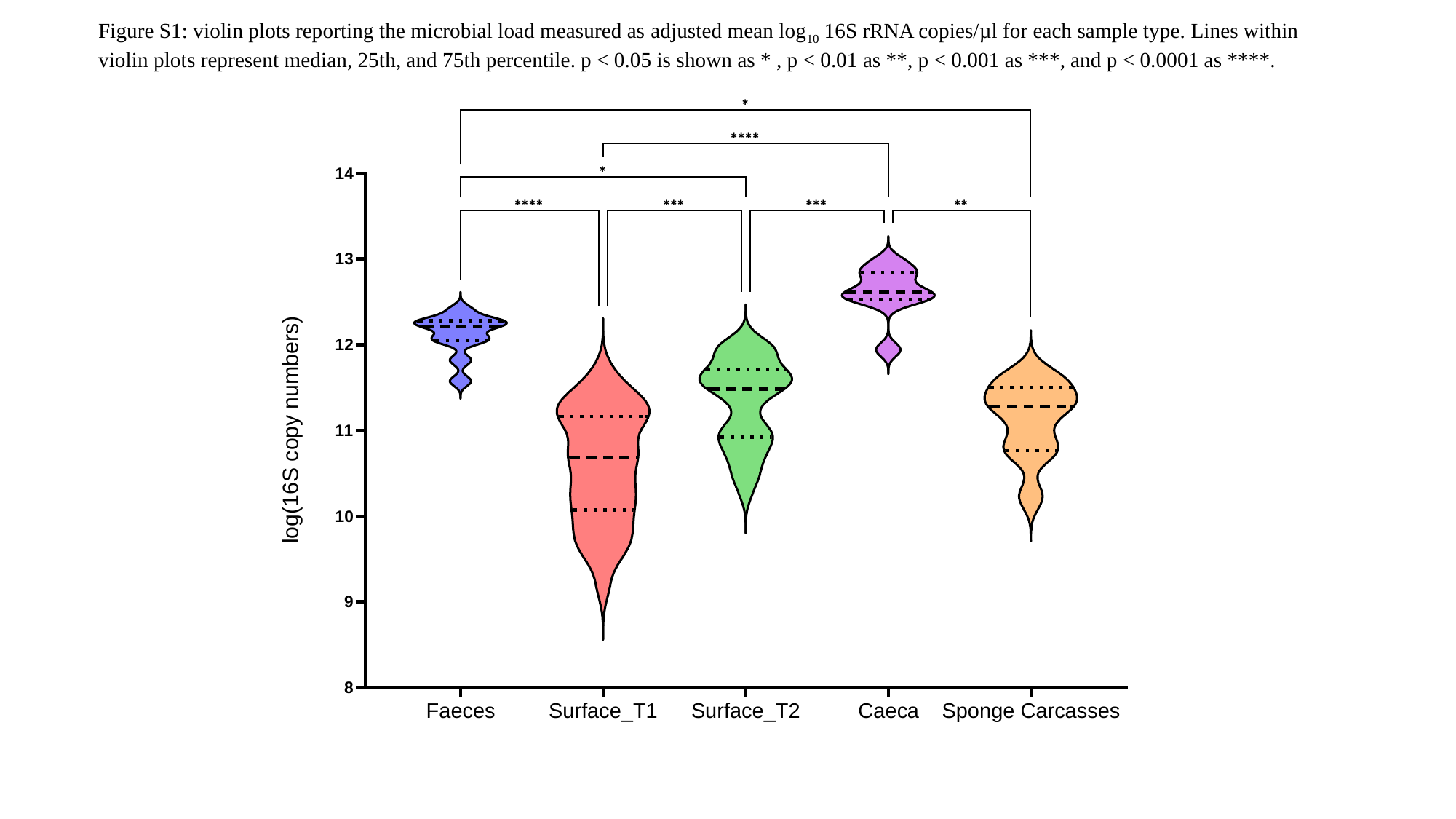

Figure S1: violin plots reporting the microbial load measured as adjusted mean log10 16S rRNA copies/µl for each sample type. Lines within
violin plots represent median, 25th, and 75th percentile. p < 0.05 is shown as * , p < 0.01 as **, p < 0.001 as ***, and p < 0.0001 as ****.
