# Supplementary material for: From Farm to Slaughter: Tracing Antimicrobial Resistance in a Poultry Short Food Chain
Source: Antibiotics (Basel). 2025 Jun 13;14(6):604. doi: 10.3390/antibiotics14060604 (PMC12190163; doi:10.3390/antibiotics14060604)
Supplement: Supplementary file 1 [file antibiotics-14-00604-s001.zip › Figure S2.pptx]

## Slide 1
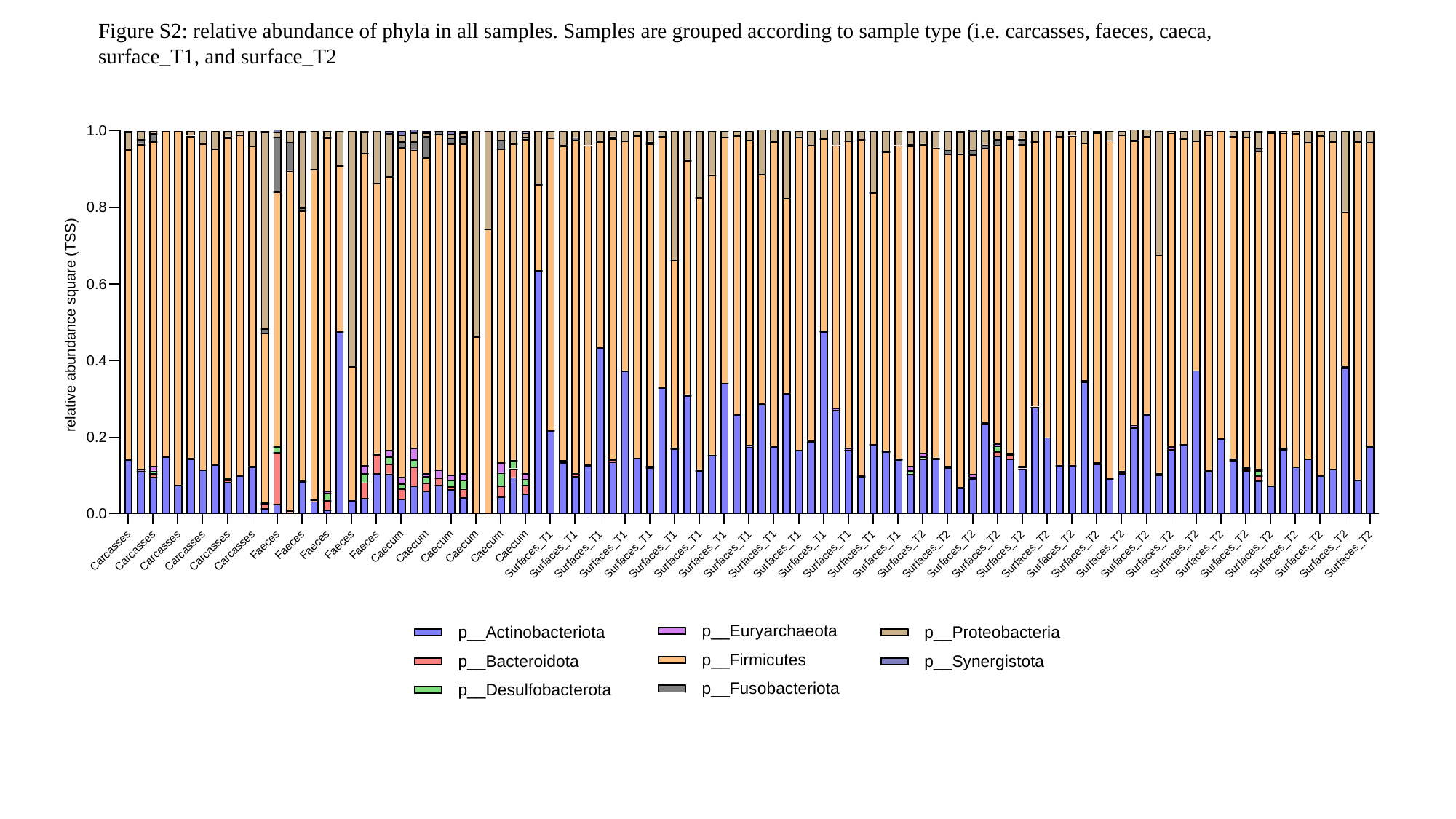

Figure S2: relative abundance of phyla in all samples. Samples are grouped according to sample type (i.e. carcasses, faeces, caeca, surface_T1, and surface_T2
