# Supplementary material for: From Farm to Slaughter: Tracing Antimicrobial Resistance in a Poultry Short Food Chain
Source: Antibiotics (Basel). 2025 Jun 13;14(6):604. doi: 10.3390/antibiotics14060604 (PMC12190163; doi:10.3390/antibiotics14060604)
Supplement: Supplementary file 1 [file antibiotics-14-00604-s001.zip › Figure S3.pptx]

## Slide 1
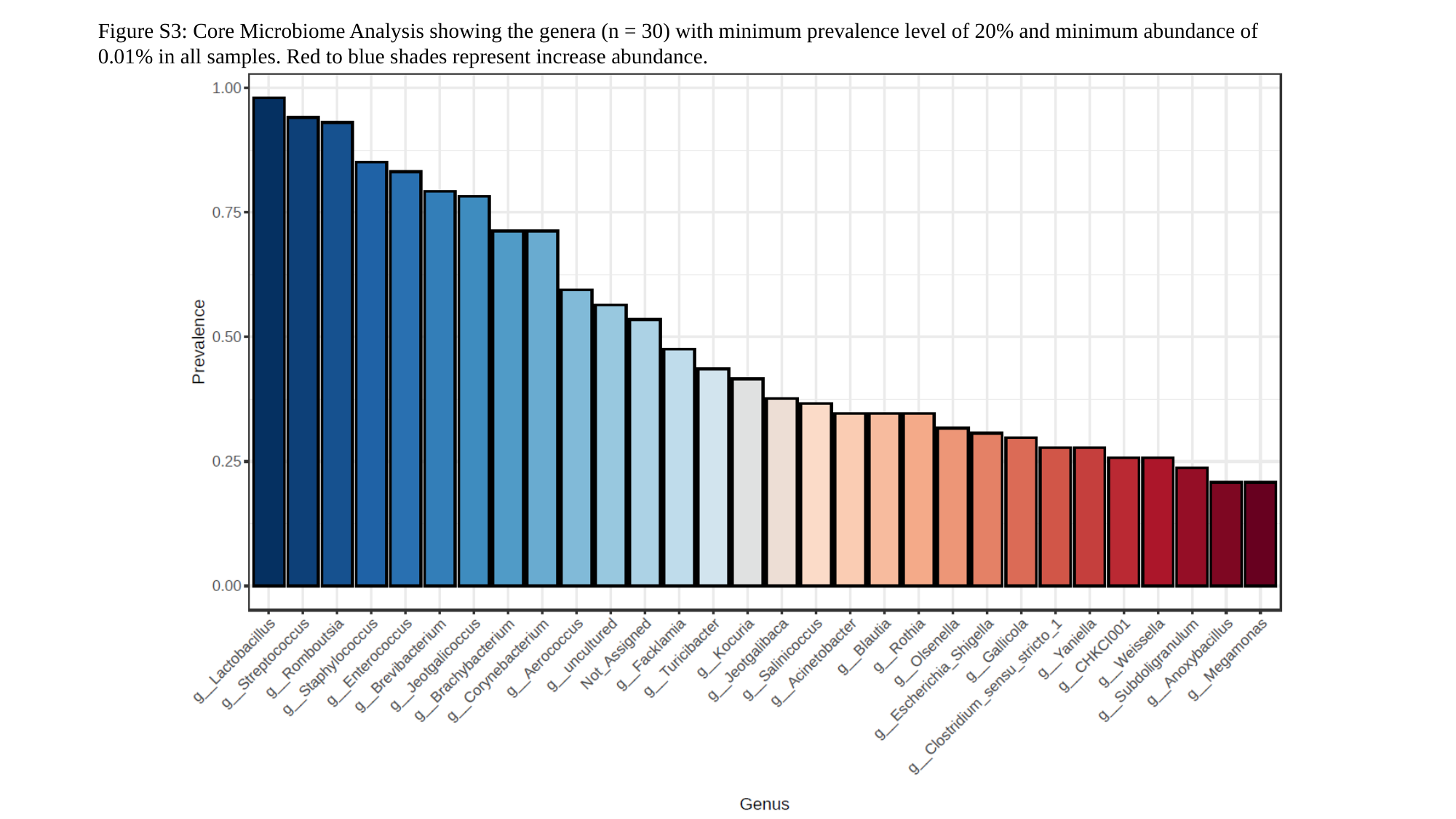

Figure S3: Core Microbiome Analysis showing the genera (n = 30) with minimum prevalence level of 20% and minimum abundance of 0.01% in all samples. Red to blue shades represent increase abundance.
