# Supplementary material for: From Farm to Slaughter: Tracing Antimicrobial Resistance in a Poultry Short Food Chain
Source: Antibiotics (Basel). 2025 Jun 13;14(6):604. doi: 10.3390/antibiotics14060604 (PMC12190163; doi:10.3390/antibiotics14060604)
Supplement: Supplementary file 1 [file antibiotics-14-00604-s001.zip › Figure S4.pptx]

## Slide 1
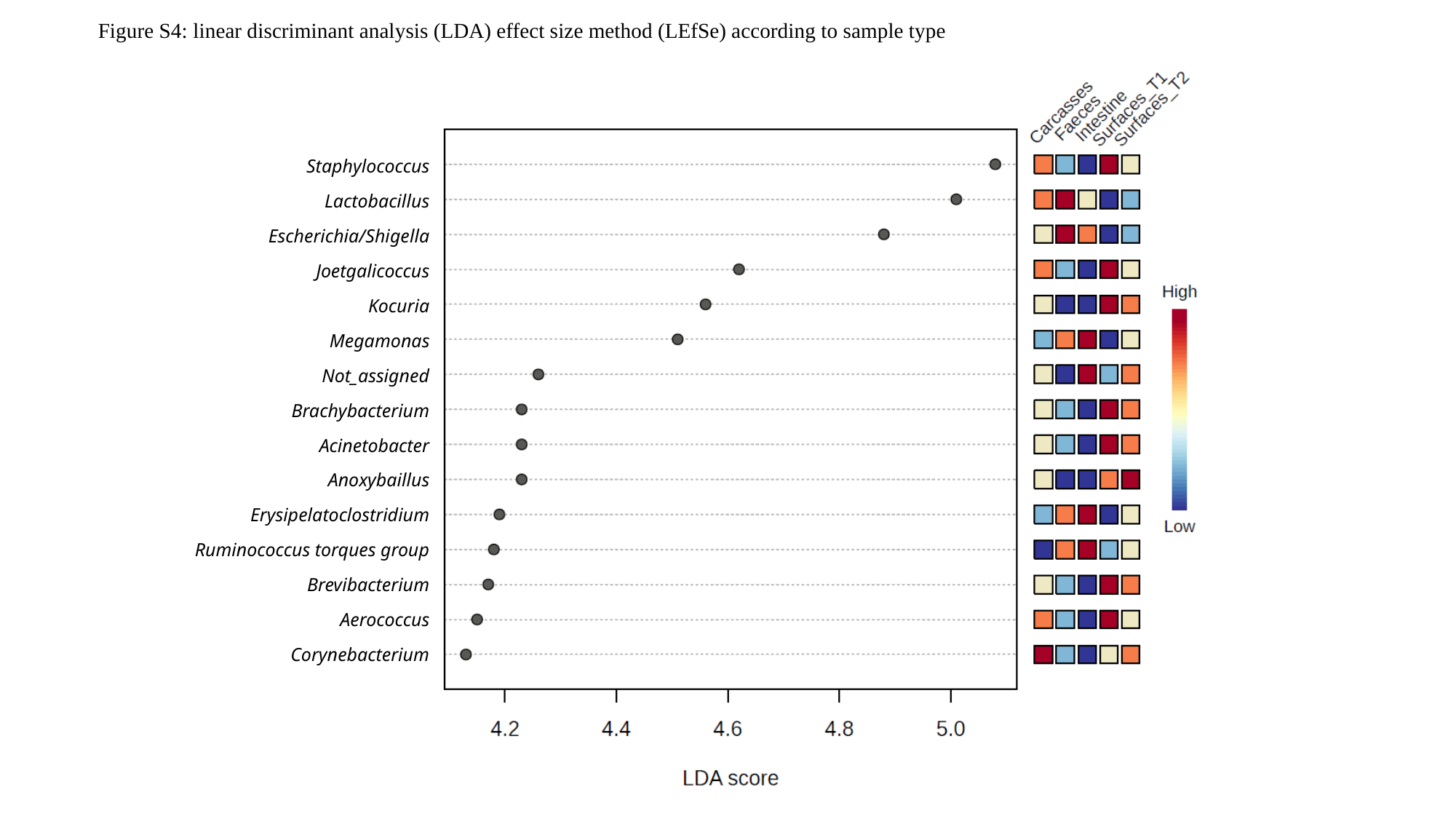

Figure S4: linear discriminant analysis (LDA) effect size method (LEfSe) according to sample type
Staphylococcus
Lactobacillus
Escherichia/Shigella
Joetgalicoccus
Kocuria
Megamonas
Not_assigned
Brachybacterium
Acinetobacter
Anoxybaillus
Erysipelatoclostridium
Ruminococcus torques group
Brevibacterium
Aerococcus
Corynebacterium
