# Supplementary material for: From Farm to Slaughter: Tracing Antimicrobial Resistance in a Poultry Short Food Chain
Source: Antibiotics (Basel). 2025 Jun 13;14(6):604. doi: 10.3390/antibiotics14060604 (PMC12190163; doi:10.3390/antibiotics14060604)
Supplement: Supplementary file 1 [file antibiotics-14-00604-s001.zip › FileS1.pdf]

Attachment A

Data Collection and Sample Accompaniment Form

FORM no. ....

Date of 1st Sampling (Farm) ..../........... /...........

Date of 2nd Sampling (Slaughterhouse)......./........... /...........

FARM INFORMATION

ADDRESS ....,.... No. .... MUNICIPALITY: .... PROVINCE: .... ULSS: ....

Farm code ....

Farm type ☐ Multi-species farm (species: ....)

☐ Single-species farm (species: ....)

Year of construction:.....................

No. of units per farm:..................... Total surface area: .... sqm

Barn type (e.g., tent structure, masonry structure, etc.): ....

No. of animals per farm: ....

Sampled barn: .... No. of animals raised per cycle: ....

Date of housing: ....

Cycle duration: from .... days to .... days

Free-range area ☐ Yes ☐ No Surface area: .... sqm

VENTILATION, FEEDING, AND WATER SUPPLY

Ventilation: ☐ natural ☐ force (comments:..)

Feed: ☐ Feed mill ☐ self-produced (comments:..)

Feed distribution: ☐ manual ☐ automatic (comments:..)

Water: ▪ water supply network ▪ well (comments:..)

Water distribution: ▪ manual ▪ automatic (comments:..)

SAMPLES

Feces: ………… (sampling points: …………)

SECTION TO BE FILLED-IN DURING SLAUGHTERHOUSE VISIT

Same group of animals as sampled on the farm: YES □ NO □

No. of slaughtered animals (from sampled farm): **……………………**

Slaughtered animal species (from sampled farm):**…………………………………………………………………………………………**

Slaughter of animals from other farms on the same day? YES □ NO □

If yes:

Total number of batches slaughtered: ………………

Animal species slaughtered: ……………………………………………………………………………

Total number of farms contributing batches: …………………

Total number of batches slaughtered before the sampled farm: ………………

Cleaning procedures: □ between slaughters □ start of the day □ end of the day □ other frequencies (specify:……………………………**.**)

Products used……

Disinfection procedures: ▪ between slaughters ▪ start of the day ▪ end of the day ▪ other frequencies (specify:……………………………**.**)

Products used ……

PRE-SLAUGHTER SAMPLES (n = 4)

- □ Sponge from conveyor belt (hooks)
- □ Sponge from water tank
- □ Sponge from plucking machine
- □ Sponge from carcass handling trolley

POST-SLAUGHTER SAMPLES (n = 6)

- □ Sponge from conveyor belt (hooks)
- □ Sponge from water tank
- □ Sponge from plucking machine
- □ Caeca (n = 10)
- □ Sponge from carcass handling trolley
- □ Sponge from carcasses (breast area, 10 carcasses)

NOTES:……

………
